# Endoplasmic Reticulum: The Favorite Intracellular Niche for Viral Replication and Assembly

**DOI:** 10.3390/v8060160

**Published:** 2016-06-07

**Authors:** Inés Romero-Brey, Ralf Bartenschlager

**Affiliations:** 1 Department of Infectious Diseases, Molecular Virology, Heidelberg University, Im Neuenheimer Feld 345, Heidelberg 69120, Germany; 2 German Center for Infection Research, Heidelberg University, Heidelberg 69120, Germany

**Keywords:** intracellular organelles, cell membranes, membrane rearrangements, nuclear envelope, peripheral endoplasmic reticulum (ER), virus-host interactions, viral replication, virion assembly, vesicles, electron microscopy

## Abstract

The endoplasmic reticulum (ER) is the largest intracellular organelle. It forms a complex network of continuous sheets and tubules, extending from the nuclear envelope (NE) to the plasma membrane. This network is frequently perturbed by positive-strand RNA viruses utilizing the ER to create membranous replication factories (RFs), where amplification of their genomes occurs. In addition, many enveloped viruses assemble progeny virions in association with ER membranes, and viruses replicating in the nucleus need to overcome the NE barrier, requiring transient changes of the NE morphology. This review first summarizes some key aspects of ER morphology and then focuses on the exploitation of the ER by viruses for the sake of promoting the different steps of their replication cycles.

## 1. Introduction

Upon infection of a host cell, viruses frequently induce alterations of intracellular organelles which serve multiple purposes, including the formation of replication factories (RFs) or the assembly of infectious virus progeny. The endoplasmic reticulum (ER) is the largest cellular organelle and the most commonly hijacked “niche” during viral infection. In this review we briefly summarize our current knowledge about the ER architecture and its functions and then discuss how viruses exploit ER membranes to promote the different steps of the viral replication cycle.

## 2. The Endoplasmic Reticulum (ER)

### 2.1. ER Morphology

**Figure 1:**
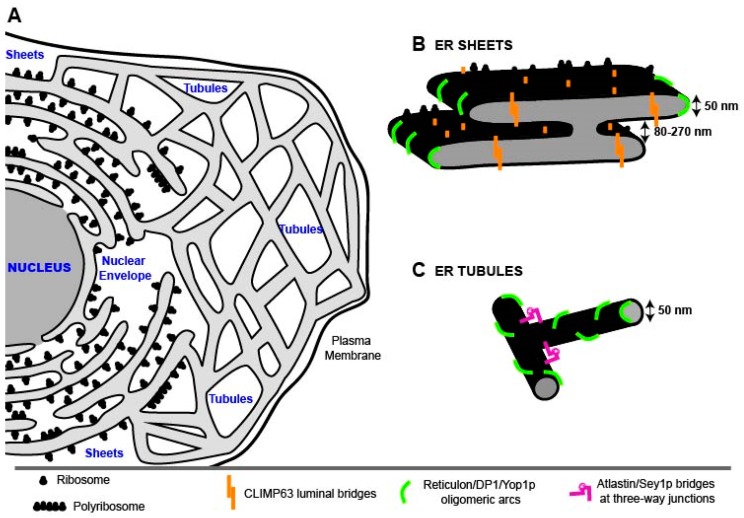
Schematic representation of the endoplasmic reticulum (ER) network organization. (**A**) The ER network comprises the nuclear envelope (NE) and the peripheral ER. The peripheral ER is composed of stacked sheets, studded with ribosomes, and tubules extending the reticular network to the plasma membrane; (**B**) Schematic of an ER sheet, depicting its multidimensional properties and the ER-shaping proteins that are responsible for the characteristic, flattened morphology. CLIMP63 is a coiled-coil protein forming oligomers bridging across the luminal space of the sheets that are also studded with ribosomes. High membrane curvature at the edges of the sheets is stabilized by reticulon/DP1/Yop1p proteins, forming wedges inside the bilayer and arc-like scaffolds around the edge. Sheets are also connected to neighboring sheets by membranous twists (“Parking garage model”, [7]); (**C**) Schematic of two ER tubules. Reticulon/DP1/Yop1p oligomers might not only determine the size of the sheet edges, but also the diameter of the tubules. These are interconnected through proteins such as atlastins/Sey1p residing at three-way junctions. The models in (B) and (C) are adapted from [8,9]. Dimensions of ER sheets and tubules refer to those found in mammalian cells.

The ER was one of the last large cell organelles to be discovered [1]. More than 100 years ago, Emilio Veratti, a student of Camillo Golgi, described a new subcellular structure, distinct from the Golgi apparatus [2] already known at that time. Fifty years later, this new organelle was first visualized through electron microscopy (EM) by Keith Porter, who observed a complex, lace-like pattern of fine strands that he designated “endoplasmic reticulum” [3]. Further EM studies by George Palade, together with Keith Porter, revealed the intricate structure of this tubular membrane network [4,5]. The ER is a highly dynamic cytoplasmic membrane system composed of two structurally distinct subdomains: the nuclear envelope (NE), enclosing the nucleus, and a polygonal network called the peripheral ER, composed of branched flattened sheets and tubules [6] (Figure 1). In the following sections we briefly summarize the morphologies and properties of these ER subdomains.

#### 2.1.1. Nuclear Envelope (NE)

The nuclear envelope consists of two flat ER membrane bilayers, the inner nuclear membrane (INM) and the outer nuclear membrane (ONM). The NE morphology is maintained via protein linkers within the perinuclear space (PNS), keeping a constant distance of 50 nm between the two membrane layers [10,11]. In addition, interactions of the INM proteins with chromatin and the lamin and of the ONM with nuclear pores and the cytoskeleton (reviewed in [12,13,14]) are required for the maintenance of the NE structure. This double membrane separates the intranuclear space from the cytoplasmic compartment and is only interrupted by nuclear pores, composed of nuclear pore complexes (NPCs) responsible for nucleocytoplasmic transport. Molecules up to 9 nm in diameter can passively diffuse through their inner channel [15], while molecules up to 39 nm in diameter in most cases require active nuclear import/export. Efficient translocation is carried out by transport receptors (karyopherins) that recognize cargos carrying specific recognition signals: nuclear localization and nuclear export signals (NLS and NES, respectively) [16].

#### 2.1.2. ER Sheets

ER sheets are a series of stacked flat cisternae which, save for the edges, have low membrane curvature [8,17,18] and can be studded with ribosomes [19,20] (Figure 1B). The structure of sheets is governed by integral membrane proteins such as CLIMP63 (cytoskeleton-linking membrane protein of 63 kDa; formally p63), which is a type II integral membrane protein [21,22,23], composed of an extended luminal coiled-coil domain, a transmembrane (TM) domain and an N-terminal cytoplasmic segment that can bind microtubules [21,24]. CLIMP63 forms oligomers via interactions between the charged coiled-coil domain that might act as a bridge between the two flat membrane sheets, thus creating a constant sheet luminal distance of 50 nm in animal cells [8,20]. Indeed, CLIMP63 depletion reduces the luminal spacing of the sheets to 30 nm, similar to the spacing observed in cell types that do not express CLIMP63, such as yeast [8].

Ribosomes and large polyribosomes preferentially localize to ER sheets. The high density of ribosomes (~1000 ribosomes/μm^2^) on ER sheets [20] might regulate their shape. Furthermore, ER membrane-bound microtubules may stabilize the rough ER by physically limiting the lateral mobility of polysomes [25,26]. Thus, two microtubule-binding proteins are enriched in ER sheets, CLIMP63 [26] and p180, which also bind ribosomes to create a platform for high-rate protein synthesis [27,28,29]. While ribosome and microtubule binding might stabilize the low-curvature ER membrane sheets, their edges owe their high curvature to the presence of wedge-shaped proteins, called reticulons (RTNs) that are also involved in shaping ER tubules [8] (described below).

A recent publication resolved for the first time the long-standing question how rough ER sheets are stacked [7]. In contrast to Golgi cisternae that are connected through protein bridges [30], ER sheets are connected by a motif that is characterized by a twisted flat membrane surface. The 3D structure is a continuous membrane system resembling a “parking garage” in which the different levels are connected by helicoidal ramps. This architecture allows an efficient and energetically favorable packing of a maximum amount of membrane sheets. This is especially important for “professional” secretory cells that have a particular requirement for membrane-bound polysomes, where the proteins to be secreted have to be synthesized. Furthermore, the variability of the helical pitch and of the corresponding distance between sheets allows the cell to adjust to different demands of its secretory capacity: when more secretory proteins are needed, more membranes can be packed into the same space by decreasing the distance between sheets. Thus, in neurons, sheets are stacked with an average distance of ~270 nm, while in cells of the salivary gland this distance is on average ~84 nm [7].

#### 2.1.3. ER Tubules

Tubules are highly dynamic structures, continually forming and rearranging and interconnecting at three-way junctions, resulting in loosely polygonal arrays that are spread throughout the cytoplasm (Figure 1C). They radiate from the NE and from ER sheets and interconnect all together, reaching various parts of the cells [31]. Tubules differ from sheets mainly by their higher membrane curvature, with a higher surface-to-volume ratio in comparison to sheets. Furthermore, tubules have a lower ribosome density than sheets and are built mainly of smooth ER membranes [19]. A shared feature of sheets and tubules, however, is their luminal space [8,20].

As for sheets, tubules are also shaped mainly by membrane-associated proteins that are involved in tubule formation and maintenance, as well as in branch point generation. Among them, RTNs, a family of highly conserved hairpin-forming membrane proteins, play a critical role in structurally shaping ER tubules [32,33,34,35,36,37]. In fact, depletion of RTN proteins reduces ER tubules and increases peripheral ER sheets, indicating that RTNs alone are sufficient to control ER tubule levels [33,36,38]. As stated above, RTNs are also found at the edges of ER sheets, generating highly curved membranes, but are excluded from flat ER regions and the NE [33]. The membrane-sculpting properties of RTNs rely on their topology, comprising two unusually short membrane-associated hairpins [9,33,39], and their ability to form immobile oligomers in the ER membrane [35] that might determine tubule diameter [6], as well as the width of the sheets. Yeast cells have two RTN genes (RTN1 and RTN2), each encoding for a single protein, whereas mammals have four (RTN1, RTN2, RTN3, RTN4/Nogo) [32,40,41,42,43,44,45] encoding eleven isoforms. Different RTN isoforms might be specialized for distinct trafficking functions [6].

Another hairpin-forming membrane protein, DP1 (defective in polyposis 1), also known as TB2 or REEP5 (receptor expression enhancing protein 5), as well as the yeast ortholog Yop1p (yeast homolog of the polyposis locus protein 1) is also involved in maintaining ER tubule structure [33]. Like RTNs, these proteins form homo-oligomers that are predicted to form arc-like structures [34,35], stabilizing high membrane curvature. Mathematical calculations support the idea that cylindrical tubules can be generated and maintained when only ~10% of the surface of the ER tubules is occupied by hairpin-forming membrane proteins [34].

Although very dynamic, ER tubules always remain continuous. Proteins with other functions, including nucleases and GTPases, play a crucial role in the formation of this continuous network. For example, dynamin-like integral membrane GTPases such as atlastins/Sey1p (synthetic enhancer of Yop1 protein) interact with the tubule-shaping proteins and enable tubule interconnections via homotypic ER-ER fusion [46,47]. Interestingly, the structure of atlastins resembles that of the mitofusins, another family of large GTPases promoting the fusion of neighboring mitochondria [48,49], with the C–termini exposed to the cytoplasm. The current fusion model assumes that atlastins regulate ER shape in a GTP-dependent manner. Fusion occurs upon dimerization of two atlastins residing in neighboring membranes. Dimerization occurs via interaction between their N-terminal GTPases, which are bound to GDP. Conversion of GDP to GTP generates conformational changes in the atlastins, pulling both ER membranes together until they fuse [50,51].

Two ER-enriched Rab GTPases (Rab10 and Rab18) were found recently to be involved in regulating ER dynamics (reviewed in [6]) and ER-ER tubule fusion ([52,53], respectively). Their role in determining the ER shape has been suggested to occur via their interaction with molecular motors (e.g., kinesin-1 and dynein) during ER sliding that would facilitate rapid ER-ER fusion [54].

### 2.2. ER Functions

The ER plays a crucial role in synthesis, modification and transport of secretory and membrane proteins, and is the site for the biosynthesis, processing and transport of several lipids (recently reviewed in [55]). In addition, the ER regulates the intracellular calcium level and forms specialized regions such as sites for vesicular export (ERES, ER exit sites) as well as contact areas with other membrane-bound organelles [52], including mitochondria [56], endosomes [57,58], peroxisomes [59], lipid droplets [60,61], phagophores [62] and the plasma membrane [20,63,64,65,66].

ER domains adopt distinct morphologies likely reflecting different functions. For instance, owing to their high curvature, tubules are better suited for surface-dependent functions, such as vesicular transport and inter-organelle signaling, whereas ER sheets immobilize polysomes on their flattened membranes and are thus specialized in protein synthesis, including protein translocation through the ER membrane, proteolytic processing, protein folding and secretion [19,33]. In fact, it has been reported that the translocation machinery is more enriched on ER sheets than in tubules [31]. The flat ER sheets might also be more stable platforms than tubules, which would allow them to better support the bulky membrane-bound polyribosomes required for protein synthesis [6]. Alternatively, differences in the morphologies of tubules and sheets might contribute to different distribution of proteins in the ER, generating bulk flow movements that would favor the retention of luminal proteins within the sheets, while membrane-bound proteins would be retained in ER tubules [6]. Moreover, ER tubules might also be involved in lipid synthesis [6]. For instance, it is known that phosphatidylserine is synthesized at ER regions called MAMs (mitochondria-associated membranes) [67] that might have a tubular morphology.

The ER is a highly dynamic organelle changing its organization in relation to multiple conditions such as the cell cycle [68,69]. In fact, during division of mammalian cells the ER undergoes spatial reorganization and a sheet-to-tubule transformation, starting with intact or fenestrated sheets (interphase ER) and changing into structures resembling tubular networks (mitotic ER). This transformation is accompanied by a reduction in membrane-bound ribosomes [68] and its extent varies between different cell lines [69]. Tubulation of the ER network might provide a simple, yet effective, mechanism for partitioning of the ER during mitosis [68]. Similar changes of the ER structure were observed upon ribosomal stripping and inhibition of RNA translation, suggesting that tubulation might be linked to translation activity on ER membranes [68]. Consistently, the level of the ribosome-binding ER protein p180 is reduced prior to cell division and knockdown of p180 expression leads to reduced ribosome density on ER membranes [28] and to fenestrated ER sheets [68]. Furthermore, overexpression of RTN4a eliminated sheet structures from the interphase ER and created long, non-branching tubules arguing that RTN abundance at the onset of cell division might contribute to the loss of sheets from the mitotic ER [68].

Depending on the cell type, the ER can adopt a wide range of organizations to adapt to different functions. Thus, the sheet-to-tubule ratio varies in different cell types reflecting the need for biosynthetic processes occurring in these two structures. In general, cells involved in synthesis and secretion of large amounts of protein (e.g., pancreatic or salivary gland cells) possess many ribosome–studded sheets [7,19], whereas poorly secreting cells (e.g., neurons, muscle cells, epithelial cells) contain an abundant tubular network [7,70].

## 3. ER Remodeling Induced upon Viral Infection

During viral infection cell membranes are co-opted for virtually every step of the viral life cycle, *i.e.*, virus entry, replication of the genome, assembly and release of virions. In many cases, cellular membranes are remodeled and utilized to build up so-called replication factories (RFs). In these cases, the membranes serve as physical support for the coordinated accumulation of viral and cellular components required for efficient replication. Moreover, membranes might ensure minimal or no exposure of viral nucleic acids to the host immune system, by shielding the viral genome from cellular pattern-recognition receptors and nucleases.

Amongst the intracellular organelles targeted by viruses for productive replication, the ER is most often usurped, resulting in a dramatic change of its morphology. In the case of viruses replicating in the nucleus, viral entry into and exit out of the nucleus results in transient modifications of the NE. Furthermore, viruses replicating in the cytoplasm, most notably positive-strand RNA viruses and a few DNA viruses, must create their replication and assembly factories, which requires extensive growth and expansion of ER membranes.

In the following sections we will describe the different strategies utilized by viruses to modify ER membranes, with a focus on viral changes affecting the peripheral ER. A comprehensive summary of virus groups remodeling this compartment is given in Table 1.

### 3.1. Transient Interactions of DNA and RNA Viruses with the Nuclear Envelope (NE)

DNA and RNA viruses replicating their genomes in the nucleus have to pass the NE (recently reviewed in [15,108,109,110,111]) to deliver the viral genome to the site of viral genome amplification [112]. In many cases, this delivery follows the classical nuclear entry pathway via the NPC. However, in some cases, the NPC can be altered. For instance, alphabaculoviruses propel the nucleocapsid through the NPC after triggering a widening of the NPC channel [113], whereas adenoviruses alter the NPC structure to release their DNA into the nucleus [114]. A much more drastic, NPC–independent nuclear entry pathway has been observed with parvoviruses [115]. These viruses utilize host cell caspases, which are proteases involved in NE breakdown during apoptosis, giving rise to 100–200 nm diameter local disruptions [116]. NE breakdown occurring during cell division is also required for most retroviruses and papillomaviruses to deliver their genome into the nucleus ([117,118], respectively).

Interestingly, polyomaviruses utilize a “detour” prior to nuclear entry via the NPC: uptake of the nucleocapsid by endocytosis, endosomal transport to the ER where the capsid is partially disassembled and transfer of the genome via the PNS into the nucleus through holes in the INM [119].

Like nuclear entry, release of newly synthesized viral genomes can also occur via the NPC as long as the size of the nucleocapsid allows NPC passage. However, the nucleocapsids of herpesviruses are too large (120 nm) to pass through the NPC [108]. Therefore, they have developed an envelopment/de-envelopment strategy to cross the NE, starting with budding of the nucleocapsid at the INM to form an enveloped particle within the PNS that is subsequently released into the cytosol by fusing with the ONM [120,121,122,123] (reviewed in [124,125]). A similar strategy is used by baculoviruses [126,127,128]. Other viruses such as polyoma- and papillomaviruses appear to leave the nucleus by disrupting the NE [129,130,131]. Although not proven, disruption or destabilization of the NE might also be used by herpesviruses, parvoviruses and adenoviruses [108].

### 3.2. Replication and Assembly of DNA Viruses at the Peripheral ER

**Figure 2:**
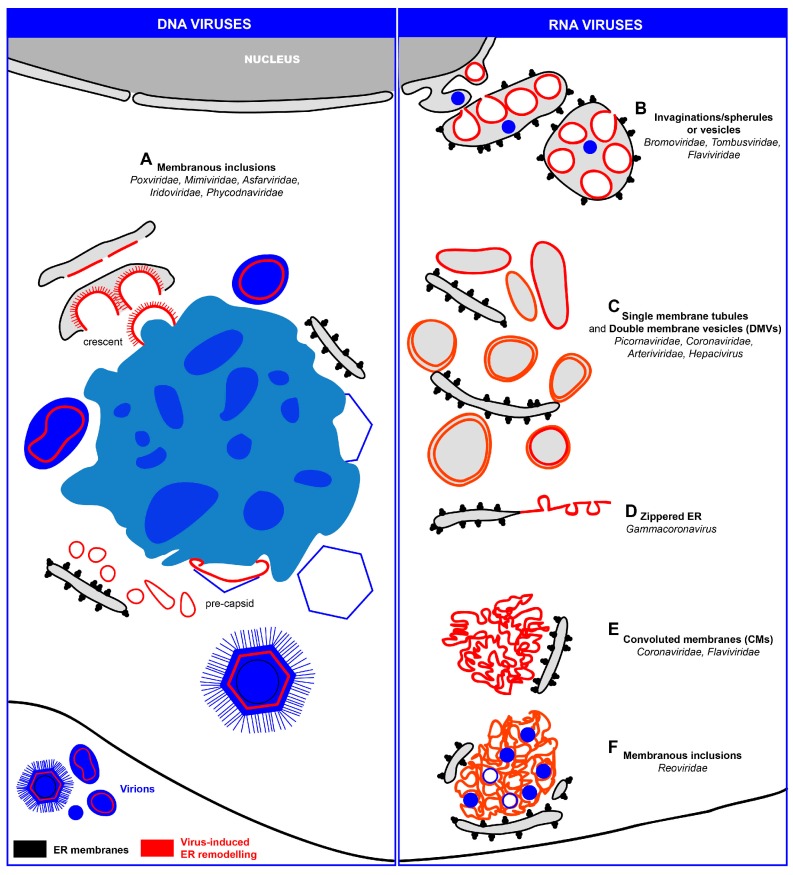
Schematic representation of ER modifications induced by DNA and RNA viruses (left and right panel, respectively). (**A**) Some DNA viruses (e.g., *Poxviridae*, *Mimiviridae*, *Asfarviridae, Iridoviridae* and *Phycodnaviridae*) replicate their genomes in the cytoplasm in close association with ER-derived membranes that are part of the replication and assembly factory. The single internal membrane bilayer of these viruses is generated through an unusual mechanism, comprising the generation of membrane sheets by rupture of ER cisternae in close proximity to the viral factories. There the single bilayers interact with capsid proteins to generate crescents and eventually pre-capsids. These factories have an ordered organization, with a preference to accumulate the viral genomes in the factory center to facilitate continuous generation of virus progeny. The only exception known so far is PBCV-1 (not shown) that accumulates the viral genome at the periphery, while the single-membrane bilayers localize to the center of the viral factory; (**B**) Some RNA viruses replicate their genomes in spherules (families *Bromoviridae* and *Tombusviridae*) or vesicles (family *Flaviviridae*), that are formed as invaginations towards the ER lumen; (**C**) Replication factories originating from “exvaginations” from the ER, giving rise to large single-membrane tubules or double-membrane vesicles (DMVs) (e.g., several members of the families *Picornaviridae*, *Coronaviridae, Arteriviriridae, Hepacivirus*); (**D**) Infectious bronchitis virus (IBV, genus *Gammacoronavirus*) induces mainly zippered ER membranes comprising tethered spherules. IBV also induces DMVs, albeit to low numbers; (**E**) ER-derived convoluted membranes (CMs) are also found in cells infected with members of the *Coronaviridae* and *Flaviviridae*. Since these structures are devoid of ribosomes, they are most likely modified smooth ER membranes; (**F**) In reovirus-infected cells, filled and empty virions (*Reoviridae*) are found embedded in a membranous inclusion of cellular origin (also called “viroplasm”) and closely surrounded by rough ER cisternae. Both ER elements and virus particles are in contact with the cytosolic face of the plasma membrane. Note that for reasons of simplicity, non-ER intracellular organelles are not shown in this schematic.

**Figure 3:**
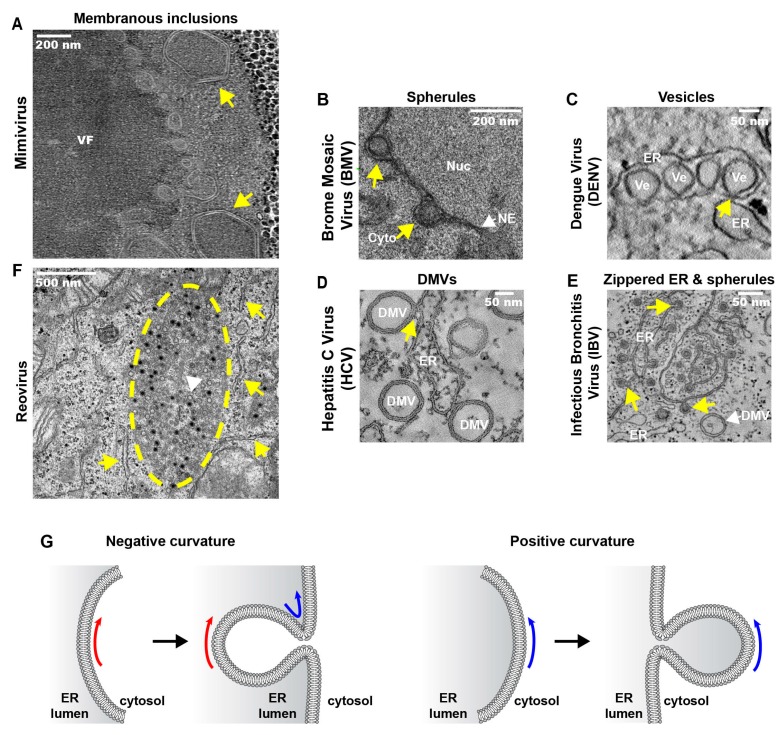
Representative electron micrographs of specialized virus-induced ER modifications. (**A**) Cytoplasmic viral factory (VF) of *Acanthamoeba polyphaga* mimivirus, 8 hpi, showing icosahedral capsids (yellow arrows) at the factory periphery; (**B**) The replication factor 1A of brome mosaic virus (BMV) localizes to outer perinuclear ER membranes in yeast cells, where it induces the formation of luminal spherules (yellow arrows) (60–80 nm) between the nucleoplasm (Nuc) and the cytoplasm (Cyto); (**C**) Dengue virus (DENV) induces the formation of invaginations of ER membranes, generating arrays of vesicles (Ve) that are known as vesicle packets (VPs). These vesicles remain connected to the cytosol through 10 nm diameter pores (yellow arrow). Shown are Huh7 cells 24 hpi; (**D**) DMVs are abundantly found in Huh 7.5 cells infected with hepatitis C virus (HCV), 16 hpi. Some of them remain connected to their source organelle, the ER (yellow arrow); (**E**) Zippered ER membranes are found in CK cells infected with the gammacoronavirus infectious bronchitis virus (IBV), 16 hpi. The zippered membranes fold to form individual spherules with a channel connecting the cytosol (yellow arrows). DMVs are also found, but to a lesser extent; (**F**) Membranous inclusions (yellow dashed line) found in HeLa cells infected with the reovirus strain T3-T1M1, 12 hpi. Rough ER cisternae (yellow arrows) are found surrounding the inclusions that contain many virions and coated microtubuli inside (white arrowhead); (**G**) Schematic representation of membrane curvature types. Negative membrane curvature results in the formation of invaginations towards the ER lumen, generating spherules or luminal vesicles. Positive membrane curvature generates exvaginations of ER membranes towards the cytosol, giving rise to cytoplasmic vesicles, tubules or DMVs. Electron micrographs are reproduced with permission from [76] (**A**), [150] (**B**), [146] (**C**,**D**) [107] (**E**) and [80] (**F**).

Nucleocytoplasmic large DNA viruses (NCLDVs) are a group of dsDNA viruses encompassing the families *Poxviridae*, *Mimiviridae, Asfarviridae, Iridoviridae* and *Phycodnaviridae* [132]. These viruses replicate their DNA partly or entirely in the cytoplasm of infected cells where they induce massive membrane rearrangements giving rise to nucleus-like structures designated viral factories (VFs) (Figure 2A and Figure 3A; Table 1). These elaborate structures enable a spatio-temporal coordination of viral replication and assembly of new virions.

One of the most critical processes is formation of inner virion membranes that are present in all NCLDVs. This is best studied, but most controversially discussed, for vaccinia virus (VACV, family *Poxviridae*) (reviewed in [123]). The absence of an obvious membrane continuity between a cellular organelle and the viral membrane led to the idea of *de novo* formation of the crescent membrane (the precursor of the inner virion membrane) [133]. Alternatively, crescent membranes might originate from the ER-Golgi intermediate compartment (ERGIC) [134,135,136,137]. Smooth ER membranes labeled with protein disulfide isomerase (PDI) and viral proteins were found close to crescents arguing that they originate from the ER [71,72,73,74,75]. Thus, the current model proposes that fragmentation of the ER membrane precedes crescent formation. As a result, crescents consisting of a single-membrane bilayer, derived from fragmented ER, and an external protein scaffold are formed towards the ER lumen, implying that the outer surface of the virion is derived from the luminal side of the ER membrane (reviewed in [138]). This is in agreement with previous data showing that, late in infection, the ER around the viral factories disassembles, coinciding with a dramatic decrease of DNA synthesis and the formation of virion precursors [139].

Likewise, the mimivirus *Acanthamoeba polyphaga* (family *Mimiviridae*) acquires its inner membrane from open membrane intermediates that correspond to crescents and accumulate at the periphery of the cytoplasmic VF [76]. Membrane biogenesis is initiated by fusion of multiple ER–derived vesicles (~70 nm diameter) allowing continuous lipid supply to the membrane-assembly zone. The resulting membrane structures subsequently rupture to form large, open, single-layered membrane sheets from which viral membranes are generated. The visualization of cell membranes studded with ribosomes at the periphery of the VF from which the vesicles seem to bud and the fact that mimivirus infection is accompanied by massive redistribution of ER markers suggest that mimivirus membranes are derived from the ER, but this remains to be confirmed. A similar process might operate in the case of the African swine fever virus (ASFV) (family *Asfarviridae*) [77].

Iridoviruses like frog virus 3 (FV3) and Singapore grouper iridovirus (SGIV, genus *Ranavirus*) replicate their genome via a similar mechanism ([140,141], respectively) whereas assembly follows a distinct pathway. The assembly site of these viruses, also known as “viromatrix,” is surrounded by mitochondria and presumably ER-derived linear and circular membranous structures [78]. These viral assembly sites have channels connecting to the nucleus possibly to facilitate the transport of newly synthesized DNA from the nucleus to the assembly site [78].

In sharp contrast to these NCLDV factories, the viral factories of *Paramecium bursaria* chlorella virus 1 (PBCV-1, family *Phycodnaviridae*) [79] are very different. While the central region consists of a network of single-membrane bilayers acting as scaffold for capsid formation, the viral genomes localize to the periphery of the factories and are excluded from the membrane-containing cores. As for ASFV [142], DNA replication of PBCV-1 is supposed to occur in the nucleus [143,144,145] prior to induction of nucleus-derived rough cisternae at the ONM. These cisternae serve as precursors for viral membranes and are recruited to the VFs, where they are ruptured into a dense network of open, single-bilayer membrane sheets accumulating in the center of the PBCV-1 factories. These open sheets interact with the capsid protein to form pre-capsids [79].

Taken together, these data reveal a common principle of all members of the NCLDV clade, the formation of an internal viral envelope during their assembly, originating at the VFs by a mechanism involving ruptured ER membranes.

### 3.3. Replication and Assembly of Positive-Strand RNA Viruses in Association with ER Membranes

The best known virus-induced ER modifications are those generated upon infection with positive-strand RNA viruses (reviewed in [146]). Numerous members of this virus group remodel ER membranes to form their replication factories (RFs) (Figure 2B–E and Figure 3B–E; Table 1). In the following sections, we will discuss the biogenesis of these factories according to their morphologies.

#### 3.3.1. ER Invaginations

A strategy frequently used by positive-strand RNA viruses to remodel ER membranes is the induction of membrane invaginations towards the ER lumen (Figure 2B). For instance, many plant positive-strand RNA viruses use ER membranes to build up RFs, named spherules (families *Bromoviridae* and *Tombusviridae*) or vesicles (family *Flaviviridae)*. One well-studied example is brome mosaic virus (BMV, family *Bromoviridae*) replicating its RNA on ER-derived spherules (~60 nm diameter) in close proximity to the nucleus [82,83] (Figure 3B). Another bromovirus, cowpea chlorotic mottle virus (CCMV), also induces the formation of spherules in the lumen of the perinuclear and peripheral ER [81]. This is also the case of red clover necrosis mosaic virus (RCNMV, *Tombusviridae*) that also induces invaginations at the NE [84]. Another example is beet black scorch virus (BBSV; *Tombusviridae*) inducing distinct cytopathological changes typical of ER aggregation and vesiculation in infected cells [85]. Three-dimensional electron tomographic analysis revealed the formation of multiple ER-originated vesicle packets (VPs), each VP enclosing a few to hundreds of independent spherules. Strikingly, these VPs were connected to each other via tubules, a rearrangement event that is rare among other virus-induced vesicles.

Positive-strand RNA viruses infecting animals and humans also induce ER invaginations as part of their RF. Best studied are members of the genus *Flavivirus* (family *Flaviviridae*) (Figure 2C). Vesicles with a diameter between 80–150 nm have been found in the lumen of ER sheets of mammalian cells infected with dengue virus (DENV) [90,91,92] (Figure 3C), West Nile virus (WNV) [87,88,147], tick-borne encephalitis virus (TBEV) [94], Langat virus (LGTV) [96], Murray Valley encephalitis virus (MVEV) [93], Japanese encephalitis virus (JEV) [89,148], St. Louis encephalitis virus (SLEV) [149] and yellow fever virus (YFV) [86]. Owing to their array-like alignment in ER sheets, these vesicles have also been called VPs. Moreover, because of the vesiculation of the ER lumen, the membrane sheets become dilated giving rise to swollen ER sacs.

The localization of dsRNA, a presumed replication intermediate, and viral replicase proteins in these vesicles as determined with immuno-EM argue that VPs might be the site of RNA replication (e.g., [87,92,95,96,147,151]). In addition, the presence of ~10 nm wide pores (e.g., [88,92,95]) linking the vesicle interior to the cytosol—as found also in BMV-infected cells [83]—ensures efficient exchange of cellular and viral components, including newly synthesized RNA with the cytoplasm.

Flaviviruses are arthropod-borne viruses (“arboviruses”) and thus also replicate in insect cells where they induce the same type of ER invaginations [96,152]. Apart from these vesicles, in TBEV–infected tick cells, elongated vesicles or tubules (up to 800 nm in length) were found that were much more abundant in persistently than in acutely infected cells. Of note, these tubules were only occasionally observed in TBEV-infected or DENV-infected mammalian cells [92]. The function of these tubules is not known, but the absence of pores suggests that the tubules might represent aberrant RFs resulting from incorrect membrane remodeling or represent an antiviral cellular defense mechanism restricting infection [96].

Based on available data we can assume that all members of the genus *Flavivirus* utilize the ER as membrane source for the formation of their RFs, whereas assembly of progeny virions seems to occur via budding into the ER lumen in close proximity to the VPs [92]. Whereas in the case of DENV, progeny virions accumulate in the ER sacs, in the case of TBEV, particles were observed in the same compartment where replication takes place [95], thus creating an optimized membranous environment to coordinate viral replication and assembly.

Structures morphologically reminiscent to those induced by flaviviruses were also found in cells infected with pestiviruses (*Flaviviridae* family). This includes dilated ER cisternae and tubules [153,154,155,156], as well as virions budding into the ER [157]. However, the precise site of RNA replication remains to be determined. Vacuoles enclosing vesicles of various sizes have been also found in bovine viral diarrhea virus (BVDV)-infected cells [158]. These structures that resemble multivesicular bodies (MVBs) might represent the site of pestiviral replication. In that case, pestiviruses appear to use a different strategy to form their RF.

Remodeling of intracellular membranes that serve as building blocks of viral RFs is not limited to the ER, but might affect other intracellular organelles such as mitochondria [159,160], lysosomes [161,162], chloroplasts [163] or peroxisomes [164]. Interestingly, in the absence of peroxisomes, tombusvirus replication switches to the ER [165]. Moreover, plant potyviruses (family *Potyviridae*) sequentially recruit ER and chloroplast membranes [166]. These observations suggest that RNA viruses have remarkable flexibility with respect to the source of membranes utilized for the construction of their RF.

#### 3.3.2. ER Exvaginations: Single- and Double-Membrane Vesicles (DMVs)

In early studies conducted with poliovirus, the prototype member of the family *Picornaviridae* (genus *Enterovirus*) identified putative replication organelles with a horseshoe-like shape designated “U bodies” [167]. Subsequent studies reported elongated single-membrane vesicles or tubules [97,168] as well as double-membrane vesicles (DMVs) in poliovirus-infected cells [98,99] (Figure 2C). Two recent publications suggest that these distinct membranous structures are induced in a time-dependent manner in poliovirus and coxsackievirus B3 (CVB3)-infected cells ([169,170], respectively). Early in infection, single-membrane tubular clusters that are transformed into bigger irregularly shaped single-membrane structures (possibly corresponding to the “U bodies”) predominate. Late in infection, they are replaced by vesicles with a much more complex structure, with two lipid bilayers tightly apposed to each other, thus giving rise to the name DMVs [169]. The exponential phase of viral RNA synthesis coincides with the predominance of single-membrane tubules, suggesting that these structures are involved in viral RNA replication, consistent with previous observations localizing active replication to the outer surface of these structures [97]. However, since DMVs originate from tubules and form during the phase when viral RNA replication increases, it is very possible that DMVs might also play an active role in viral RNA synthesis. Alternatively, DMVs might represent a dead-end structure of RFs that in the course of the CVB3 replication cycle transform into complex multi-membraned vesicles [170]. In any case, the observation that in cells infected with the foot and mouth disease virus (FMDV; genus *Aphthovirus*) single- and double-membrane structures are also induced, albeit with low efficiency, suggests that the formation of both types of membrane rearrangements might be a general property of the *Picornaviridae* family members [171].

The source of membranes used by these viruses to build up their RFs is a matter of debate. Golgi–derived membranes might serve as initial site of poliovirus replication [172,173], whereas late in infection, markers of the ER, Golgi and lysosomes were all found to be associated with the poliovirus RF [97,98,168,174]. Furthermore, it is known that enterovirus infection causes a strong inhibition of the anterograde ER-Golgi transport [175,176] and treatment with Brefeldin A, a well-known inhibitor of the secretory pathway, abrogates enterovirus replication [177,178]. These findings, together with the reported role of COPII [174,179] and COPI vesicles [180], as well as other components of the secretory pathway [181,182,183] and the ERES [184] argue that membranes from multiple cellular sources, mainly from ER and Golgi, are used by picornaviruses to build up their RFs [98].

Members of the families *Arteriviridae* and *Coronaviridae*, included in the order *Nidovirales*, usurp ER membranes to generate DMVs as well (Figure 2C). These can remain connected to ER membranes via their outer membrane and are interconnected to form a reticulovesicular network. DMVs were found in cells infected with equine arterivirus (EAV) [100,101], mouse hepatitis virus (MHV) [185,186], severe acute respiratory syndrome (SARS)-coronavirus (CoV) [102,103] and Middle East respiratory syndrome coronavirus (MERS-CoV) [187]. Similar DMVs have also been found in cells infected with hepatitis C virus (HCV, family *Flaviviridae*) [104,105,106] (Figure 3D). This observation was surprising because flaviviruses (e.g., DENV and TBEV) belonging to the same family induce RFs with a very different morphology (ER membrane invaginations; see above) suggesting that even closely related viruses can utilize distinct cellular pathways to build up their RFs.

How the DMVs are formed remains enigmatic. They might originate from a single-membrane vesicle or tubule budding from its donor organelle (the ER) that might undergo secondary invagination. While this pathway would be similar to the one utilized by picornaviruses, a “pre–DMV” structure has not yet been proven to exist in the case of HCV. We note that Ferraris and colleagues reported single-membraned structures, along with DMVs, in HCV-infected cells [106], but it remains to be determined whether these single-membrane structures are precursors of DMVs.

Several lines of evidence suggest that DMVs might be the site of viral RNA replication. First, the kinetics of DMV appearance correlates with viral replication [105,186]. Second, immuno-EM studies identified dsRNA in the DMV lumen [101,103,185,188]. Third, DMVs isolated from HCV-replicating cells contain an active replicase allowing metabolic labeling of viral RNA [188]. While these findings argue for DMVs as sites of RNA replication, the exact localization of the replicase remains unclear. It might be localized on the DMV surface or in its interior. In the latter case, newly synthesized viral RNA has to be released from these double–sheltered organelles. However, DMVs found in arterivirus- and coronavirus-infected cells are not connected to the cytosol [101,103] and in the case of HCV, only ~10% of DMVs have an opening towards the cytosol [105]. These results favor the assumption that only a minority of DMVs are actively engaged in RNA replication at a given time.

#### 3.3.3. Zippered-ER, Spherules and DMVs

As described above, members of the family *Coronaviridae* induce the formation of DMVs. This is also the case for infectious bronchitis virus (IBV), an important poultry pathogen belonging to the genus *Gammacoronavirus*. However, the most striking structures induced by IBV are zippered ER membranes [107] that distinguish this virus from the members of the genus *Betacoronavirus*, e.g., MHV, SARS-CoV or MERS-CoV. The zippered ER was associated with 60–80 nm diameter spherules (Figure 2D and Figure 3E). Their interior is connected through a 4.4 nm long channel with the cytoplasm, making them the ideal sites for the synthesis of viral RNA. In contrast, DMVs might be a by-product not directly involved in RNA synthesis or corresponding to inactive remnants of active RFs [189].

#### 3.3.4. Convoluted Membranes (CMs)

Branching networks of membranes known as convoluted membranes (CMs) have been observed in cells infected with several positive-strand RNA viruses, notably members of the families *Coronaviridae* [103,186,187] and *Flaviviridae* [91,92,151,190] (Figure 2E). These structures form a continuous network with the VPs induced by DENV [92] or with the DMVs induced by SARS-CoV [103]. CMs are frequently connected with the ER [103] from which they seem to originate [92], most likely smooth ER as indicated by the lack of ribosomes. Viral and cellular proteins localize to these structures [92,103,151,186,190] and they are induced by the sole expression of individual viral proteins such as NS4A in the case of DENV [191,192].

It has been suggested that CMs might correspond to storage sites for proteins and lipids required for replication and assembly. The fact that CMs are physically linked with ER-containing invaginations and contain NS3 would be consistent with this assumption [92]. Alternatively, CMs might represent sites of polyprotein processing [151,190]. This conclusion is based primarily on the strong immunolabeling for NS2B and NS3 and the lack of NS1 and NS4B detection. Since polyprotein cleavage occurs co-translationally and thus should happen at the rough ER, this model would require the formation of rather stable processing intermediates that are transferred from the rough ER to the CMs where further cleavage would occur.

CMs appear to have a distinct lipid composition, most notably high amounts of cholesterol. Of note in cells of insects that are cholesterol auxotrophs and lack several enzymes of the cholesterol biosynthesis pathway [193], CMs are absent, which might be due to the lack of cholesterol [152].

### 3.4. Replication and Assembly dsRNA Viruses at ER-Related Inclusions

Reoviruses are non-enveloped, dsRNA viruses. Upon their entry into cells, the viral proteins form distinct inclusions (Figure 2F and Figure 3F), *i.e.*, large cytoplasmic structures, also known as “viroplasms” providing a physical scaffold to coordinate RNA replication and virion assembly. Although the inclusions were thought to be membrane-free structures, a recent study identified membranes within the inclusions that appear to be derived from ER, Golgi or from an as yet undefined cellular source [80]. Furthermore, rough ER cisternae were found in contact with reovirus inclusions [80] reminiscent to the architecture of rubella virus RFs [162]. Although the latter are derived from lysosomal membranes, the rough ER cisternae provide the framework for newly synthesized viral proteins to facilitate their incorporation into viral replication organelles [162]. A similar mechanism might also operate with reovirus factories. Alternatively, since reoviruses are non–enveloped, the ER might be used as a membranous scaffold to facilitate virus particle trafficking to the cell periphery, thus bypassing the Golgi apparatus [80]. Rotavirus, another member of the family *Reoviridae*, also induces the formation of cytoplasmic viroplasms [194]. To acquire the outer shell proteins VP4 and VP7, immature rotavirus particles have to bud in the ER to allow the assembly of mature particles [195].

## 4. Cross-Talk between Viral and ER Proteins

Only few proteins involved in the regulation of the ER shape are known to be highjacked by viral proteins to create membranous RFs, and even less is known in the case of cellular ER-shaping proteins. One example is the RTNs that are usurped by several positive-strand RNA viruses (reviewed in [196]). Interestingly, the positive-curving RTNs seem to play a role in the formation of the negative curvature invaginations induced by BMV on ER membranes [197]. Although in principle counterintuitive, RTNs might be involved in stabilizing the neck of the invaginations, which has a positive curvature (Figure 3G). Furthermore, depletion of RTNs results in the formation of smaller vesicles, thereby indicating that RTNs might be involved in partially cancelling the negative membrane curvature induced by viral proteins [196].

RTN3 has been shown to interact with proteins of enteroviruses (2C) and HCV (NS4B) that are known to induce the formation of tubules and vesicles with positive curvature ([198] and [199], respectively). While in the case of enteroviruses these interactions enhance viral replication [198], possibly by promoting the formation of positively curved tubules [196], in the case of HCV these interactions are inhibitory. In fact, RTN3 was found to bind to HCV NS4B and impair viral replication [199]. Conversely to enteroviruses, HCV does not induce membrane tubules, but primarily DMVs. Whether the antagonistic effects of RTNs for enteroviruses and HCV are related to the use of alternative cellular pathways for RF formation remains to be determined.

Another ER-resident protein that plays a critical role in the life cycle of flavi- and hepaciviruses is Rab18. This protein not only localizes on ER membranes, but is also located at lipid-droplet (LD)–associated membranes [200,201]. Both organelles, ER and LDs, play a crucial role for these virus groups with Rab18 acting as a “linker” tethering LDs to the cytosolic side of the ER. In HCV-infected cells, Rab18 interacts with HCV nonstructural protein 5A (NS5A) [202,203], which is involved both in RNA replication and virion assembly. Interestingly, the viral core protein accumulates on the surface of LDs. Thus, Rab18 is used by HCV for recruitment of LDs in close proximity to the replication sites, thereby also recruiting the capsid protein. Since HCV assembly is tightly linked to LDs, Rab18 appears to be exploited to facilitate the coordination of RNA replication and virus assembly [202,203]. Consistent with this assumption it was found that Rab18 is required for HCV particle formation [204]. In the case of DENV, Rab18 is involved in the interaction of fatty acid synthase (FASN) with NS3, contributing to the recruitment of FASN to the ER and LDs. This recruitment is thought to promote fatty acid biosynthesis, required for membrane proliferation [205].

A critical role of ER-resident proteins has also been reported for tombusvirus replication. Proteins of this virus bind to the ER resident VAMP-associated protein (VAP) [206]. These interactions likely facilitate the formation of membrane contact sites (MCS) between the ER and peroxisomal and mitochondrial membranes, where tombusviruses build up their RFs. Furthermore, these interactions also promote the recruitment of oxysterol-binding protein-related proteins (ORPs), resulting in an enrichment of sterols at the MCS, required for the formation of tombusvirus-induced spherules.

Similarly, the oxysterol-binding protein-like 1 protein (OSBP1), found at ER-Golgi MCS, plays a critical role in the formation of the rhinovirus RFs on Golgi-derived membranes, in close proximity to the ER. OSBP1 binding to the ER-resident protein VAP-A appears to trigger a phosphatidylinositol 4-phosphate (PI4P)-cholesterol flux [207] at MCSs to drive the formation of the viral RFs. This flux is mediated by several proteins: OSBP1, shuttling PI4P and cholesterol; the ER-associated PI4P phosphatase Sac1, hydrolyzing PI4P, and the phosphatidylinositol (PI) transfer protein beta (PITPb), shuttling PI. Of note, OSBP is also required for the replication of enteroviruses in general including poliovirus, coxsackievirus and enterovirus-71 [208].

Interestingly, the DMVs induced by HCV are also enriched in VAP-A and VAP-B [188]. Moreover, OSBP is required for HCV replication, too [209]. These findings suggest that HCV exploits VAPs and OSBP to remodel ER membranes by mediating lipid exchange in order to create or stabilize the RFs.

## 5. Conclusions and Future Perspectives

The morphology of cellular organelles is a fundamental question in cell biology and its elucidation has only become possible with the development of high-resolution imaging techniques, most notably EM and more recently developed 3D-EM methods (reviewed in [210]). Taking advantage of these approaches, important information on the 3D architecture of the endoplasmic reticulum (ER) has become available [20,68]. However, even though the complete ER of a cell has been visualized [69,211,212] our knowledge of the homeostasis of ER morphology and the proteins that “sculpt” the ER shape into a dynamic network of sheets and tubules is still rather limited. Gaining further insight into these aspects requires a more comprehensive identification and characterization of involved proteins. It is interesting to note that mutations in some ER-shaping proteins are linked to neurological diseases such as Alzheimer’s and hereditary spastic paraplegia (HSP) (reviewed in [6]). This example underscores how important the integrity of ER morphology is for proper cell and organ function.

Given the central role of the ER in maintenance of cellular homeostasis, it is frequently targeted by viruses to promote the individual steps of their life cycles. Yet, many aspects of how viruses usurp ER function to promote these different steps are poorly understood. For instance, double-membrane vesicles (DMVs) are the most predominant membranous structures detectable in hepatitis C virus (HCV)-infected cells. However, we still do not know how these vesicles are formed. Given their striking morphological resemblance to autophagosomes, DMV biogenesis might be linked to the autophagy machinery. Indeed, several studies reported an involvement of autophagy in the HCV life cycle [213], but neither the precise step of the viral life cycle depending on autophagy nor the molecular details of the process have been defined. It is also unclear what is the function of CMs that most likely correspond to modified smooth ER and are frequently detected in flavivirus-infected cells. For many viruses utilizing the ER and replicating in the cytoplasm, we also do not know the exact site of RNA replication. For SARS-CoV, evidence has been presented that RNA replication might occur inside closed DMVs [103]. If that is the case, the question arises how newly produced progeny RNA is released into the cytoplasm to be used for the assembly of infectious progeny virus. One possibility is a proteinaceous channel formed by viral or cellular proteins. Another possibility is that RNA replication occurs at “precursor” sites and DMVs correspond to remnants of formerly active replicases that are on their way to degradation or removal out of the cell.

Addressing these and several other questions requires a comprehensive strategy that combines biochemical and cell biological approaches with cutting-edge imaging methods, most notably correlative light and electron microscopy [214]. It is clear that combining these methods with the use of ER-dependent viruses as sensitive and targeted probes will provide new and important insights into ER biology and its exploitation by pathogens.

## Figures and Tables

**Table 1:** Virus-induced endoplasmic reticulum (ER) modifications.

Induced-ER Modification	Virus	Reference	Method Used to Detect	
			Membrane Remodeling	ER Origin
**Membranous inclusions**	Vaccinia Virus (VACV)	[71]	TEM	IF, IEM
		[72]	ET, CEMOVIS	IEM
		[73]	TEM	IF, IEM
		[74]	TEM	IF, IEM, Western blot
		[75]	TEM	IF
	*Acanthamoeba polyphaga* Mimivirus	[76]	IF, TEM, STEM-T	*
	African Swine Fever Virus (ASFV)	[77]	TEM, ET, CEMOVIS, STEM-T	Western blot
	Frog Virus 3 (FV3)	[78]	TEM, ET	*
	*Paramecium Bursaria* Chlorella Virus 1 (PBCV-1)	[79]	STEM-T, FIB-SEM	*
	Reovirus	[80]	IF, TEM	*
**Invaginations/Spherules or vesicles**	Cowpea Chlorotic Mottle Virus (CCMV)	[81]	TEM	TEM
	Brome Mosaic Virus (BMV)	[82]	n.s.	IF
		[83]	TEM	TEM
	Red Clover Necrosis Mosaic Virus (RCNMV)	[84]	Confocal microscopy	Confocal microscopy, Western blot
	Beet Black Scorch Virus (BBSV)	[85]	Confocal microscopy, TEM, ET	Confocal microscopy, IEM
	Yellow Fever Virus (YFV)	[86]	TEM	TEM
	West Nile Virus (WNV)	[87]	TEM	IF, IEM
		[88]	ET	IF
	Japanese Encephalitis Virus (JEV)	[89]	TEM	TEM
	Dengue Virus (DENV)	[90]	TEM	TEM
		[91]	TEM	TEM
		[92]	TEM, ET	IF, IEM
	Murray Valley Encephalitis Virus (MVEV)	[93]	TEM	TEM
	Tick Borne Encephalitis Virus (TBEV)	[94]	TEM	n.s.
		[95]	ET	IEM
	Langat Virus (LGTV)	[96]	TEM, ET	IF
**Single-membrane tubules and double-membrane vesicles (DMVs)**	Poliovirus type 1	[97]	TEM	IEM
		[98]	TEM	Western blot
		[99]	TEM	Density gradient fractionation
**DMVs**	Equine Arterivirus (EAV)	[100]	TEM	TEM, IF, IEM
		[101]	TEM	ET
	SARS-Coronavirus	[102]	TEM	IEM
		[103]	TEM, ET	ET
	Hepatitis C Virus (HCV)	[104,105]	TEM	Western blot
		[76]	TEM, ET	IF, ET
		[106]	TEM	n.s.
**Zippered ER**	Infectious Bronchitis Virus (IBV)	[107]	TEM, ET	TEM, ET

Plant viruses are highlighted in green. TEM, transmission electron microscopy; IF, immunofluorescence; IEM, immuno-EM; ET, electron tomography; CEMOVIS, cryo-EM of vitreous sections; STEM-T, scanning transmission electron microscopy-tomography; FIB-SEM, focus ion beam-scanning electron microscopy; * the origin from ER membranes has only been suggested; not shown (n.s.). References are given in chronological order.
